# Enrichment of gut-derived *Fusobacterium* is associated with suboptimal immune recovery in HIV-infected individuals

**DOI:** 10.1038/s41598-018-32585-x

**Published:** 2018-09-24

**Authors:** Soo Ching Lee, Ling Ling Chua, Siew Hwei Yap, Tsung Fei Khang, Chan Yoon Leng, Raja Iskandar Raja Azwa, Sharon R. Lewin, Adeeba Kamarulzaman, Yin Ling Woo, Yvonne Ai Lian Lim, P’ng Loke, Reena Rajasuriar

**Affiliations:** 1 Centre of Excellence for Research in AIDS (CERiA), University of Malaya, 50603 Kuala Lumpur, Malaysia; 2 Department of Parasitology, Faculty of Medicine, University of Malaya, 50603 Kuala Lumpur, Malaysia; 3 University Malaya Cancer Research Institute, University of Malaya, 50603 Kuala Lumpur, Malaysia; 4 Institute of Mathematical Sciences, Faculty of Science, University of Malaya, 50603 Kuala Lumpur, Malaysia; 5 University of Malaya Centre for Data Analytics, University of Malaya, 50603 Kuala Lumpur, Malaysia; 6 Department of Medicine, Faculty of Medicine, University of Malaya, 50603 Kuala Lumpur, Malaysia; 7 Peter Doherty Institute for Infection and Immunity, University of Melbourne, Melbourne, Victoria Australia; 8 Department of Infectious Diseases, Monash University and Alfred Hospital; Royal Melbourne Hospital, Melbourne, Australia; 9 Department of Obstetrics and Gynecology, Faculty of Medicine, University of Malaya, Kuala Lumpur, Malaysia; 10 Department of Microbiology and Medicine, New York University School of Medicine, New York, NY 10016 USA; 11 Department of Pharmacy, Faculty of Medicine, University of Malaya, 50603 Kuala Lumpur, Malaysia

## Abstract

We explored the gut microbiota profile among HIV-infected individuals with diverse immune recovery profiles following long-term suppressive ART and investigated the relationship between the altered bacteria with markers of immune dysfunction. The microbiota profile of rectal swabs from 26 HIV-infected individuals and 20 HIV-uninfected controls were examined. Patients were classified as suboptimal responders, sIR (n = 10, CD4 T-cell <350 cells/ul) and optimal responders, oIR (n = 16, CD4 T-cell >500 cells/ul) after a minimum of 2 years on suppressive ART. Canonical correlation analysis(CCA) and multiple regression modelling were used to explore the association between fecal bacterial taxa abundance and immunological profiles in optimal and suboptimal responders. We found *Fusobacterium* was significantly enriched among the HIV-infected and the sIR group. CCA results showed that *Fusobacterium* abundance was negatively correlated with CD4 T-cell counts, but positively correlated with CD4 T-cell activation and CD4 Tregs. Multiple linear regression analysis adjusted for age, baseline CD4 T-cell count, antibiotic exposure and MSM status indicated that higher *Fusobacterium* relative abundance was independently associated with poorer CD4 T-cell recovery following ART. Enrichment of *Fusobacterium* was associated with reduced immune recovery and persistent immune dysfunction following ART. Modulating the abundance of this bacterial taxa in the gut may be a viable intervention to improve immune reconstitution in our setting.

## Introduction

The gastrointestinal (GI) tract is recognised as a crucial site for the pathogenesis of HIV infection. During acute stages of HIV infection, there is a significant and rapid depletion of CD4+ T-cells from the GI tract, leading to persistent mucosal dysfunction even after years of suppressive antiretroviral therapy (ART)^1–6^. Poor reconstitution of the gut-associated lymphoid tissue (GALT) and epithelial barrier dysfunction following ART have been attributed to the continued presence and replication of HIV, as well as cytomegalovirus (CMV) in mucosal tissues^7–10^. The disruption of gut integrity (both immunologic and structural) promotes persistent translocation of bacterial products into the peripheral circulation, a phenomenon which has been associated with systemic immune activation^11^ and mortality in HIV^12^.

In addition to microbial translocation-mediated immune activation, the composition of intestinal bacteria and its metabolites may also play a key role in HIV immunopathogenesis^13–15^. The diversity and composition of gut commensals are altered in men who have sex with men (MSM)^16^ with HIV-infection and also following the commencement of ART^17,18^. This HIV-associated dysbiosis, assessed from fecal and mucosal-associated samples, has been shown to correlate with markers of immune activation including blood and colonic CD4 and CD8 T-cell activation markers^15,19,20^, levels of hsCRP^15^, sCD14^15,17–19,21,22^, TNFalpha^21,22^ and IL-6^21^. Interventional studies in HIV and SIV-infection have suggested that modulation of gut microbiota using dietary pro/prebiotics may be a viable approach to reduce HIV-related immune activation^23–25^. On the other hand, a recent meta-analysis on the impact of probiotics on CD4 T-cell counts reported limited benefits^26^. More recently, fecal microbial transplant (FMT) was used as a strategy to modulate gut microbiota in HIV^27^. In that study, only limited engraftment was found in ART-suppressed individuals following FMT, while no change was seen in systemic immune activation levels 8 weeks post-FMT. This result contradicts an earlier report of favorable FMT outcome in SIV-infected macaques^28^. The mixed results suggest gaps in our understanding of the complex interaction between commensal gut bacteria, HIV infection and its pathogenesis.

More than 95% of the global HIV epidemic occurs in developing countries where socio-economic status and environmental factors such as diet and endemic parasitic infections may significantly influence the composition of gut commensals^29,30^. Yet to date, only a few studies outside of the USA and Europe have examined how differences in gut microbiota may influence HIV immunopathogenesis^31–33^. To this end, our objectives were to explore the changes in gut microbiota profile of individuals in Malaysia (a middle income country) with diverse immune recovery outcomes following long-term suppressive ART, and explore the influence of altered bacterial taxa on markers of immune activation and CD4 T-cell composition following ART.

## Results

### Characteristics of study population

Table 1 summarises the demographic and clinical characteristics of the 26 HIV-infected and 20 HIV uninfected controls who participated in this study. Among the HIV-infected participants, 62% (16/26) were classified oIR and 38% (10/26) sIR. Participants in the sIR arm were older than oIR and uninfected controls (median (IQR): 49 (41–52) years, 40 (34–45) years and 31 (28–46) years, respectively). All participants were males. There was no significant difference in the proportion of MSM in the HIV-infected (optimal and suboptimal) and uninfected control groups (p > 0.05 for all paired comparisons). Apart from expected significant differences in baseline and current CD4 T-cell counts as well as CD4:CD8 ratio between the oIR and sIR groups, all other HIV-related clinical variables and hepatitis B/C co-infection status were comparable in the two groups.

**Table 1:** Baseline demographic and clinical characteristics of the study subjects (N = 46).

Characteristic	Uninfected (n = 20)	HIV optimal Immune Recovery (n = 16)	HIV suboptimal Immune Recovery (n = 10)	p value^a^	
				Between HIV-infected vs uninfected	Between HIV optimal vs suboptimal
Age, years (IQR)	31 (28–46)	40 (34–45)	49 (41–52)	0.052^*MW*^	0.045^*MW*^
Ethnicity, n (%)				0.618^*F*^	0.206^*F*^
Chinese	11 (55.0)	9 (56.3)	9 (90.0)	—	—
Malay	6 (30.0)	4 (25.0)	1 (10.0)	—	—
Indian	3 (15.0)	3 (18.8)	0 (0.0)		
Gender, n (%)					
Male	20 (100.0)	16 (100.0)	10 (100.0)	—	—
MSM, n (%)	11 (55.0)	10 (62.5)	4 (40.0)	1.000^*F*^	0.422^*F*^
Smoking history, n (%)	8 (40.0)	9 (56.3)	6 (60.0)	0.373^*CS*^	1.000^*F*^
History of ADIs, n (%)	—	10 (62.5)	9 (90.0)	—	0.190^*F*^
ART Regimen, n (%)					
NNRTI-based regimen	—	16 (100.0)	10 (100.0)	—	—
Duration of ART, years (IQR)	—	4 (3–7)	5 (3–7)	—	0.729^*MW*^
Antibiotic intake at sampling, n (%)	—	0 (0.0)	3 (30.0)	—	0.046^*F*^
Hepatitis C co-infection, n (%)	—	—	—	—	—
Hepatitis B co-infection, n (%)	—	1 (6.3)	0 (0.0)	—	1.000^*F*^
Baseline CD4^+^ T-cell count, cells/μL, (IQR)	—	213 (156–383)	29 (23–99)	—	0.001^*MW*^
Current CD4^+^ T-cell count, cells/μL, (IQR)	—	726 (656–1031)	272 (262–338)	—	<0.001^*MW*^
Current CD4/CD8 ratio, (IQR)	—	0.74 (0.54–0.96)	0.38 (0.33–0.44)	—	<0.001 ^*MW*^
Immunological profile					
% Naïve CD4 T-cells, (IQR)	41(31–49)	37 (27–55)	26 (13–29)	0.018^*MW*^	0.035^*MW*^
% Central Memory CD4 T-cells, (IQR)	36 (27–39)	36 (34–40)	44 (40–47)	0.047^*MW*^	0.055^*MW*^
% Effector Memory CD4 T-cells, (IQR)	16 (12–22)	19 (12–26)	29 (21–37)	0.062^*MW*^	0.052^*MW*^
% TDEM CD4 T-cells, (IQR)	2 (1–9)	3 (2–8)	1 (1–2)	0.465^*MW*^	0.101^*MW*^
% CD4 T-cell senescence (CD28-CD57+), (IQR)	3 (0–9)	8 (2–15)	3 (0–4)	0.411^*MW*^	0.177^*MW*^
% CD8 T-cell senescence (CD28-CD57+), (IQR)	30 (11–40)	28 (22–43)	34 (28–54)	0.185^*MW*^	0.216^*MW*^
% CD4 T-cell activation (CD38 + HLA-DR+), (IQR)	4 (4–7)	4 (4–7)	8 (6–9)	0.560^*MW*^	0.012^*MW*^
% CD8 T-cell activation (CD38 + HLA-DR+), (IQR)	15 (11–23)	13 (10–19)	19 (14–24)	0.900^*MW*^	0.071^*MW*^
% CD4 Tregs, (IQR)	6 (5–7)	5 (5–6)	8 (7–9)	0.819^*MW*^	0.002^*MW*^
Plasma sCD14 (X10^6^), pg/mL, (IQR)	1.83 (1.47–1.98)	1.72 (1.61–2.04)	1.92 (1.71–2.45)	0.637^*MW*^	0.336^*MW*^
Plasma kynurenine/tryptophan ratio, (IQR)	0.024 (0.022–0.027)	0.028 (0.023–0032)	0.029 (0.027–0.033)	0.041^*MW*^	0.585^*MW*^

IQR – Results were presented in median (interquartile range, IQR), n (%), n = number of subjects.

^a^Variables are significantly different between groups if p < 0.05 using *T* = Students’s t test, *MW* = Mann Whitney test, *CS* = Chi-square tests or *F* = Fisher’s exact test.

Abbreviations; ADI-AIDS-defining illness; TDEM- Terminally differentiated effector memory; MSM- Men who have sex with men; ART- Antiretroviral therapy; Treg – T regulatory cells.

### Different microbial profile in HIV optimal, suboptimal and uninfected controls

**Figure 1:**
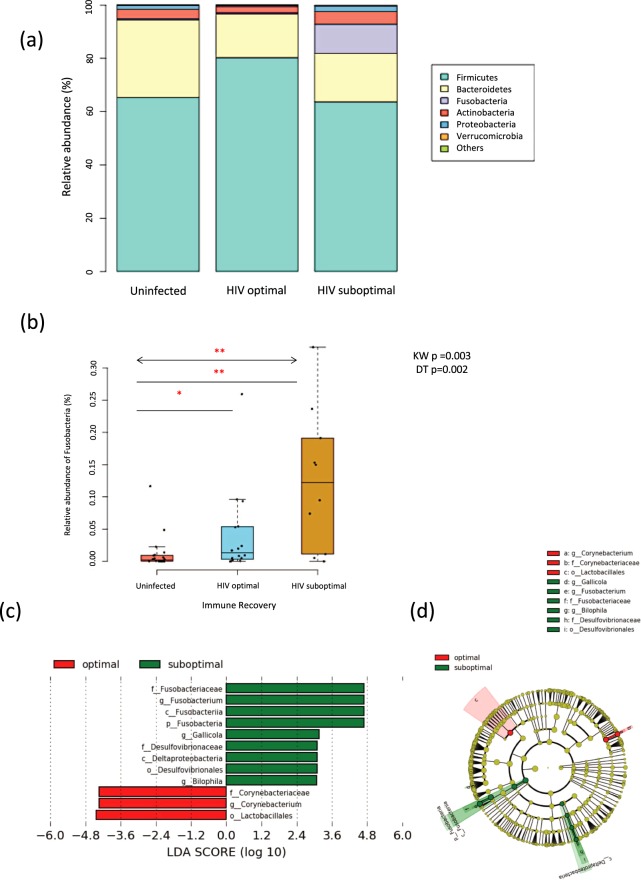
Overview of the gut microbiota profile in HIV-infected (i.e. HIV optimal and HIV suboptimal) and uninfected groups. (**a**) The stacked bar represents the median relative abundance of the most dominant gut microbiota phyla in the uninfected, HIV optimal and HIV suboptimal groups. (**b**) Box-dot plot comparing the relative abundances of Fusobacteria among the uninfected, HIV optimal and HIV suboptimal groups. **p < 0.005 and *p < 0.05, the arrowed line represents Kruskal-Wallis test with Dunn’s post-hoc testing while the straight line represents Mann-Whitney U test. (**c**) LEfSe was used to compare gut microbiota between HIV optimal and HIV suboptimal groups. The HIV suboptimal-enriched taxa are displayed with a positive LDA score (green) while the HIV optimal-enriched taxa are shown with a negative LDA score (red). With a log LDA score above 3.00, we found an increased abundance of OTUs contributed by Fusobacteriaceae, *Gallicola* and *Bilophila* among HIV suboptimal subjects, while HIV optimal subjects had increased abundance of Lactobacillales and *Corynebacterium*. All results shown were tested by Kruskal-Wallis and adjusted with the post Benjamini-Hochberg correction for multiple testing. (**d**) Taxonomic cladogram derived from LEfSe analysis of 16S sequences. OTUs showing significant difference between groups are shaded either red (HIV optimal enriched taxa) or green (HIV suboptimal enriched taxa).

Illumina Miseq sequencing at the V4 gene region of bacterial 16S rRNA resulted in 2,756,344 high quality reads with an average of 59,920 ± 106,599 reads per subject. The profile of the most dominant taxa was similar in both the uninfected and the HIV optimal groups, with an abundance of Firmicutes (median relative abundance: 65.3% vs. 80.3%, p = 0.886), followed by Bacteroidetes (29.2% vs. 16.4%, p = 0.364) and Actinobacteria (3.5% vs. 2.4%, p = 0.494) (Fig. 1a). However, in the sIR arm, there was a dominance of Fusobacteria (11.0%), which was not obvious in the other two arms. Further analysis of this bacterial taxa in the entire cohort showed a significant increase in the relative abundance of Fusobacteria in participants from the sIR compared to oIR and uninfected controls (Fig. 1b; Kruskal-Wallis test (KW): p = 0.003 with Dunn’s test: p = 0.002), implying the potential importance of this taxa in the pathogenesis of suboptimal CD4 T-cell reconstitution following ART in this cohort. We note that there was a wide variability in the relative abundance of *Fusobacterium* within the suboptimal group. However, we could not find any obvious association between any of the measured clinical features or biomarkers in our cohort for this sub-structuring. The relative abundance of phyla and genus in each subject is shown in Supplementary Fig. S2.

Although the microbial composition between the HIV-infected and uninfected groups differed significantly as indicated by beta diversity using unweighted UniFrac distance metric (p < 0.05, pseudo-*F* statistic = 1.55; Supplementary Fig. S3a and b), alpha diversity, which measures species richness and evenness, was however similar between these groups (p > 0.05). When assessed by immune recovery status, no significant differences in both beta and alpha diversity were found (p > 0.05 for both; Supplementary Fig. S3c and d, Supplementary Fig. S4a–c).

### Alteration of specific bacterial taxa in relation to HIV-infection and immune recovery status

The LEfSe analysis identified several differentially abundant microbial taxa (Supplementary Fig. S5a and b; Supplementary Table S1) in the HIV-infected and uninfected groups which included the phylum Fusobacteria and its associated taxonomic levels: class Fusobacteria, order Fusobacteriales, family Fusobacteriaceae and genus *Fusobacterium*. There was also a notable increase in the abundance of the family Veilonellaceae^14,34^ and a depletion of the genus *Eubacterium*^21,35^, as previously described in other HIV studies.

We further analysed the differences in the abundance of microbiota between oIR and sIR individuals. The phylum Fusobacteria and its descendant genus *Fusobacterium* were again enriched in the sIR group (Fig. 1c,d; Supplementary Table S3) while the bacterial taxa belonging to the family Corynebacteriaceae and its genus *Corynebacterium* were significantly depleted in the sIR arm together with the order Lactobacillales. We also compared the relative abundance of *Fusobacterium* in the different immune recovery groups against the overall mean of the relative abundance of *Fusobacterium* measured in the whole cohort (overall mean relative abundance = 0.0458). In this analysis, we found that the majority of sIR (70.0%; 7/10) had a higher relative abundance of *Fusobacterium*, whilst only 25.0% (4/16) and 10.0% (2/20) of the oIR and uninfected groups, respectively displayed an increase in the relative abundance of *Fusobacterium*.

To better understand the relative importance of the different bacterial taxa in modulating CD4 T-cell recovery, we restricted the subsequent analysis to bacteria which were found altered at the lowest taxonomic level and with a LDA score threshold of 3.0 in the LEfSe approach (order Lactobacillales, genus *Fusobacterium*, *Gallicola*, *Bilophila*, and *Corynebacterium)*.

### Associations between the altered bacterial taxa and markers of immune dysfunction

**Figure 2:**
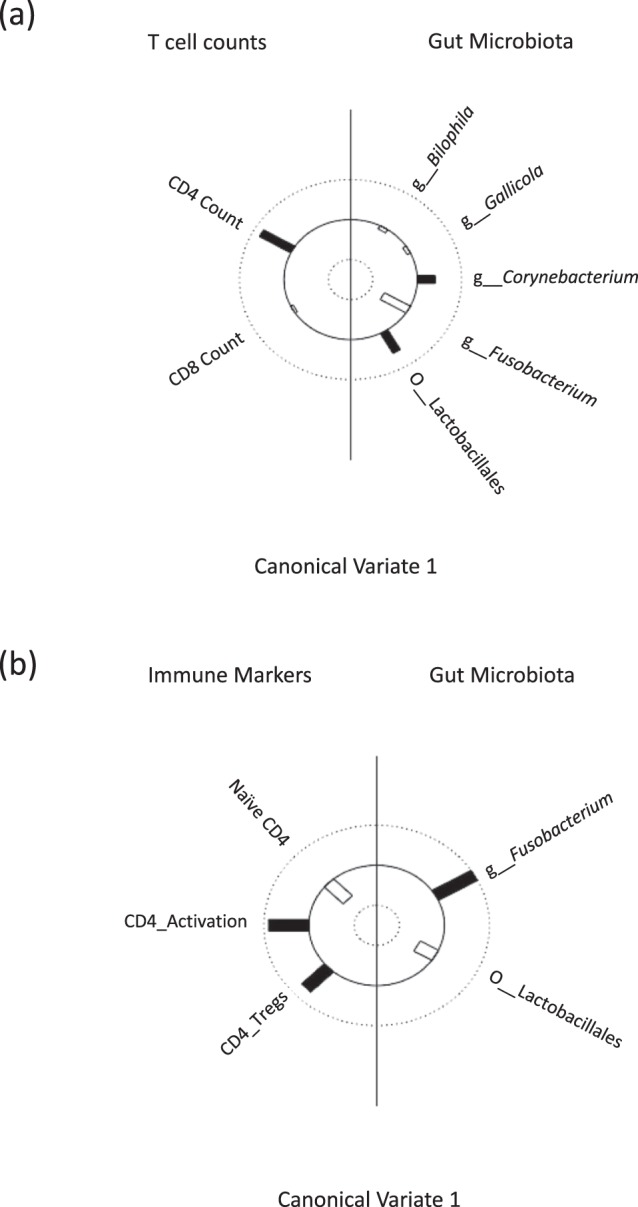
The association between CD4 T-cell counts and markers of immune dysfunction with selected bacterial taxa that differ significantly in relative abundance between the optimal and the suboptimal responders as identified from LEfSe analysis. Helio plot of the canonical correlation analysis (CCA) computed for (**a**) CD4 and CD8 T-cell counts and (**b**) markers of immune dysfunction in HIV-infected individuals. This can be interpreted as linear correlation coefficients between the Y-variables (CD4 and CD8 T-cell counts or markers of immune dysfunction) on the left side and the X-variables (selected bacterial taxa) used as predictor on the right side. The height of the bar is proportional to the magnitude of the canonical loading, while filled bars represent a positive association and open bars a negative association.

CCA results suggested that CD4 T-cell count (Canonical loading, CL = 0.846) was positively correlated with the relative abundance of *Corynebacterium* (CL = 0.437) and Lactobacillales (CL = 0.582), but inversely correlated with the relative abundance of *Fusobacterium* (CL = −0.691) (Fig. 2a; Supplementary Table S3). Lactobacillales and *Fusobacterium* had the strongest correlations with CD4 T-cell counts (CL > 0.50 for both taxa). CD4 T-cell count was negatively correlated with *Fusobacterium* relative abundance (p = 0.002, R = −0.58) (Supplementary Fig. S5c) but not with *Corynebacterium* and Lactobacillales relative abundance (data not shown), thus implying a more complex relationship between CD4 T-cell count and these bacterial taxa. We further analysed the association between the relative abundance of Lactobacillales and *Fusobacterium* on CD4 T-cell counts using multiple linear regression models while adjusting for selected confounders including baseline CD4 T-cell counts, age, antibiotics exposure and MSM status. We found that *Fusobacterium* ($\hat{\beta }$ = −0.64, p = 0.001) was significantly associated with poorer CD4 T-cell recovery following ART (Table 2).

**Table 2:** Multiple linear regression models assessing the role of selected bacterial taxa (*Fusobacterium* and Lactobacillales) in modulating CD4 T-cell counts in HIV-infected participants.

Models	Coefficients	Standard error	t	95% Confidence Interval		p-value
				Lower bound	Upper bound	
Model 1						
Intercept	14.10	10.60	1.33	−8.02	36.22	0.199
g__*Fusobacterium*	−0.64	0.16	−3.90	−0.98	−0.30	0.001
Baseline CD4 T-cell count	0.01	0.01	0.10	−0.01	0.03	0.331
Age	−0.27	0.15	−1.81	−0.60	0.04	0.086
Antibiotic exposure	6.89	4.02	1.71	−1.50	15.29	0.102
MSM	3.55	2.50	1.42	−1.67	8.77	0.171
R^2^ = 0.64						
Model 2						
Intercept	6.44	13.23	0.49	−21.15	34.03	0.632
o__Lactobacillales	0.38	0.18	2.08	−0.001	0.76	0.050
Baseline CD4 T-cell count	0.03	0.01	2.66	0.01	0.05	0.016
Age	−0.27	0.18	−1.51	−0.66	0.11	0.148
Antibiotic exposure	1.13	4.63	0.24	−8.52	10.78	0.810
MSM	4.88	3.25	1.50	−1.89	11.65	0.148
R^2^ = 0.47						
Model 3						
Intercept	10.39	10.95	0.95	−12.53	33.31	0.355
g__*Fusobacterium*	−0.57	0.17	−3.25	−0.93	−0.20	0.004
o__Lactobacillales	0.19	0.16	1.19	−0.14	0.52	0.250
Baseline CD4 T-cell count	0.01	0.01	1.25	−0.01	0.03	0.228
Age	−0.28	0.15	−1.84	−0.59	0.04	0.082
Antibiotic exposure	5.86	4.08	1.44	−2.67	14.39	0.166
MSM	4.74	2.67	1.77	−0.86	10.33	0.092
R^2^ = 0.66						

Abbreviations: MSM-men who have sex with men.

Using CCA, we also explored the association between Lactobacillales and *Fusobacterium* relative abundance with various markers of immune dysfunction found to be significantly different in the optimal and suboptimal arm in our cohort; the proportion of naïve CD4 T-cells, CD4 T-cell activation and CD4 Tregs (Table 1). In this analysis, we observed that CD4 T-cell activation (CL = −0.923) and CD4 Tregs (CL = −0.639) were positively correlated with an increase in the abundance of *Fusobacterium* (CL = −0.951) but negatively correlated with *Lactobacillus* (CL = 0.361) (Fig. 2b; Supplementary Table S4). Additionally, the proportion of naïve CD4 T-cells (CL = 0.509) was negatively correlated with *Fusobacterium* (CL = −0.951) but positively with the relative abundance of *Lactobacillus* (CL = 0.361). Thus, microbial dysbiosis in HIV may be associated with the immunopathogenesis of suboptimal CD4 T-cell recovery observed in some individuals despite long-term suppressive ART.

## Discussion

The relationship between gut microbiota and immune recovery following long-term suppressive ART is still poorly understood. This is the first study to explore this association in a developing country setting, where environmental factors that differ from those in high income countries may influence the gut commensal community composition differently. In this study, we showed that specific alterations in bacterial taxa composition occur in individuals with HIV-infection, and in individuals who experience suboptimal immune recovery (sIR) following ART. Poor immune reconstitution was most strongly associated with an increased abundance of *Fusobacterium*, even after adjusting for clinical co-variates previously shown to independently influence immune reconstitution and gut microbiota composition. Additionally, increased relative abundance of *Fusobacterium* was also significantly correlated with reduced naïve CD4 T-cells, increased CD4 T-cell activation and increased regulatory T-cells measured in peripheral blood, all markers of persistent immune dysfunction in HIV infection. These data suggest that changes in gut microbiota composition specifically the enrichment of *Fusobacterium*, may contribute to the immunopathogenesis of poor CD4 T-cell reconstitution following ART in our setting.

Consistent with prior reports of altered gut microbiota profile in HIV-infected individuals^15,21,32^, we also found an increase in the abundance of bacteria from the families of Fusobacteriaceae, Succinivibrionaceae and Micoplasmataceae in the HIV-infected compared to the uninfected group^14,15,21,35–37^. When HIV-infected individuals were further divided according to their immune recovery profile following a minimum of 2 years of suppressive ART, there was an increased relative abundance of *Fusobacterium*, *Gallicola* and *Bilophila*, in the suboptimal group compared to the optimal recovery group. While microbial diversity has been reported to be lower in HIV-infected (vs. uninfected)^17,34,35^ and suboptimal (vs. optimal) CD4 T-cell recovery^16,17^ groups, it was not observed to be statistically significant in the present study. Beta diversity as measured using the unweighted UniFrac distance was significantly associated with HIV status, but not immune recovery. Thus, significant associations in gut microbiota composition were observed in relation to HIV infection and immune discordance following ART in our setting, consistent with previous studies^15,21,32^.

Notably, the genus *Fusobacterium* from the Fusobacteria phylum was significantly more abundant in individuals with suboptimal vs. optimal CD4 T-cell recovery as well as uninfected controls. The association between the relative abundance of this taxa and reduced CD4 T-cell counts remained statistically significant even after adjusting for the effects of age, baseline CD4 T-cell counts, MSM status and antibiotic usage. We also measured markers of immune dysfunction in the HIV-infected participants and explored the association of these cellular markers with the altered bacterial taxa in the optimal and suboptimal recovery groups. Consistent with prior studies, sub-optimal CD4 T-cell recovery in our cohort was associated with a reduction in naive T-cell subsets, increased T-cell activation and increased T regulatory cells^38^. In canonical correlation analysis, the genus *Fusobacterium* again had the strongest modulating effect on this immunological profile followed by the order Lactobacillales and the genus *Corynebacterium*. Previous cross-sectional studies reported an increased abundance of Fusobacteria in the rectal/gut and oral microbiota of individuals with untreated^35,39^ and treated HIV-infection^21^, but here, we show that abundance is also associated with the patient’s dysfunctional immune profile. In a longitudinal study among HIV-infected MSM, a significant increase in the abundance of Fusobacteria was noted as individuals developed more severe immune deficiency while on zidovudine monotherapy^37^. Our findings corroborate previous observations and further suggest that Fusobacteria enrichment in individuals receiving stable ART in a developing country setting is clinically relevant. Future studies and identification of specific strains by culture isolation or qPCR are needed to establish cause-effect relationships between these bacteria and immune function in HIV-infected individuals.

*Fusobacterium* is a butyric acid-producing bacteria. Currently, indication of its pathogenic role in HIV has largely been confined to its increased abundance in the oral cavity, and its association with HIV-associated periodontitis^40^. In non-HIV related studies, increased gut *Fusobacterium* has been associated with the pathogenicity of inflammatory disorders, including inflammatory bowel disease^41–43^, acute appendicitis^44^ and colorectal cancer^45,46^, suggesting a role in regulating local gut immunity. In mechanistic studies, *Fusobacterium* has been shown to be a strong promoter of NF-kb activation^46^ and an inhibitor of histone deacetylases (HDAC), with the potential to reactivate HIV from latently infected cells *in vivo*^47^. Thus, enrichment of *Fusobacterium* in the gut could contribute to HIV-1 re-activation of latently infected cells in GALT, further compromising the integrity of the gut mucosal barrier. *Fusobacterium* also possesses independent virulent factors that may directly induce apoptotic cell death in polymorphonuclear cells (PMNs)^48^. These pathways could lead to increased local and systemic immune activation, subsequently increasing bystander CD4 T-cell death, thus potentially explaining the inability of these individuals to achieve normalised peripheral CD4 T-cell thresholds despite years of suppressive ART. We observed higher levels of CD8 and CD4 T-cell activation in individuals with suboptimal CD4 T-cell recovery, but only the latter was correlated with the abundance of *Fusobacterium*. A high turnover of CD4 T-cells could also lead to increased homeostatic proliferation of naïve CD4 T-cells in circulation, leading to a reduced proportion of this subset in favour of expanded memory CD4 T-cell subsets, as observed in our cohort.

We also found an increase in circulating Tregs in individuals with suboptimal immune recovery, which correlated with the observed alterations in microbiota composition in this population. Both mucosal and peripheral Tregs have been found to retain their suppressive function during chronic HIV infection^49^, and have been associated with reduced HIV-specific immunity especially in individuals with low CD4 T-cell counts^50^. No study to date has described an association between *Fusobacterium* and Tregs in HIV-infected individuals, though a prior study in SIV-infected rhesus macaques has linked the depletion in gut-resident Lactobacillales to an increase in IDO (indoleamine 2,3-dioxygenase) activity^20^, which is known to promote Treg differentiation in circulation and in rectosigmoidal tissues^51^. A previously reported reduction in the abundance of Lactobacillales^18,31^ was also observed in the present cohort and associated with an increase in Tregs in canonical correlation analysis. However, reduced Lactobacillales was not correlated with increased plasma IDO activity in our study (data not shown). The lack of correlation could potentially be explained by the presence of other IDO mediators including IFN-α levels, which we recently showed to remain elevated despite years of suppressive ART in our setting^52^. The association between reduced Lactobacillales and CD4 T-cell counts was also weaker when compared to *Fusobacterium* in our cohort and was lost when both bacterial taxa were included in a multiple linear regression model. Multiple pathways may contribute to the loss of CD4 T-cells in treated HIV-infection and thus it is conceivable that several bacterial taxa may contribute to this process by different and/or inter-related mechanisms^13^. Future *in vitro* studies should further explore if there is a symbiotic relationship between the depletion of protective Lactobacillales and the enrichment of pathogenic *Fusobacterium* which could potentiate the loss of CD4 T-cells in HIV.

The strength of this study is in the characteristics of the cohort which included individuals with distinct immune recovery profiles despite years of suppressive ART. Additionally, all participants were men who received the same NNRTI-based regimen; MSM status, which has been shown to significantly affect the gut microbial composition, was recorded and accordingly adjusted for in our analysis. Several caveats necessarily accompany our interpretation of the present observational study. First, dietary patterns peculiar to the participants may have influenced the observed outcome, but no information about this aspect was obtained from the participants. Secondly, our choice of using rectal swabs with fecal material instead of biopsy samples for sampling gut microbiota composition was mainly motivated by logistical considerations, which nonetheless may potentially bias the result. Thirdly, the biological effects attributed to the microbiota found in this study are likely driven by their metabolites, the analysis of which is beyond the scope of this study. We suggest future work to include a component of metabolomics and meta-transcriptomics as data from these two lines of enquiry are essential to our understanding of the underlying mechanisms associated with altered microbiota in HIV.

In conclusion, we found the composition of the gut microbiota of individuals receiving suppressive ART to be significantly different from those without HIV-infection. Specific bacterial taxa were associated with impaired CD4 T-cell reconstitution and persistent immune dysfunction following ART. Interventions that modulate the composition of gut microbiome, particularly *Fusobacterium*, may be a useful approach to augment immune reconstitution in individuals with suboptimal CD4 T-cell recovery despite years of suppressive ART in our setting.

## Methods

### Study design and participants

Participants for this study were identified from a previous cross-sectional study established to study functional immune response following suppressive ART at University Malaya Medical Centre (UMMC)^53^. Briefly, individuals fulfilling the following inclusion criteria were recruited during their routine clinic appointments: (1) men >18 years old; (2) receiving suppressive ART (HIV RNA <50 copies/mL) for a minimum of 2 years; (3) CD4 T-cell counts persistently <350 cells/µL (suboptimal immune recovery, sIR) or >500 cells/µL (optimal immune recovery, oIR) on suppressive ART; and (4) no acute illness at recruitment. In addition, a total of 20 HIV uninfected controls were recruited from a local community-based clinic and individuals responding to study fliers.

All participants and controls provided informed consent and answered a standardised questionnaire while clinical parameters for the HIV-infected participants were retrieved from medical records. The study protocol was approved by the UMMC ethics review board (Permit Number: MEC 20161-1992). All procedures contributing to this research were conducted in accordance with the relevant guidelines and regulations.

### 16s rRNA sequencing of gut microbiota

DNA was extracted from rectal swabs as previously described^53^ and was PCR-amplified targeting the V4 region of the 16S rRNA gene using a protocol modified from Caporaso *et al*.^54^, details of which have been previously described^55^. Amplicon sequencing was subsequently performed on the Illumina MiSeq platform with a 2 × 150 cycle run (Illumina, San Diego CA, USA).

### Gut microbiota bioinformatic analysis

Quantitative Insights Into Microbial Ecology (QIIME) software version 1.9.1 was used for the pre-processing steps, and for the downstream analysis (i.e. taxa summary plot, alpha diversity and beta diversity) of gut microbiota sequencing data^56^ (details in Supplementary Methods).

To identify bacterial taxa that are statistically different between the groups of interest, Geometric Mean of Pairwise ratios (GMPR) normalization^57^ was used to overcome the sparse and compositional effects on the raw count data. Then, the LDA Effect Size (LEfSe) analysis was performed using the online interface Galaxy (https://huttenhower.sph.harvard.edu/galaxy/)^58^. The threshold for statistical significance using the Kruskal-Wallis rank sum test was set at the 5% level of significance, a threshold of 3 was used for the logarithmic LDA score. Correction for multiple testing was done using the Benjamini-Hochberg procedure^59^.

### Soluble activation markers

Whole blood was collected in Ethylenediaminetetraacetic acid (EDTA) vacutainers and processed within 4 hours of collection. Soluble CD14 was measured in plasma using the Quantikine sCD14 kit (R&D Systems, USA) according to the manufacturer’s instructions as previously described^60^. Kynurenine and tryptophan concentrations were measured by liquid chromatography-tandem mass spectrometry (LC-MS/MS, Agilent, 6400 series) as previously described^61^. The HPLC conditions used were as follows: the column was a Synergi Polar RP column (75 mm × 4.6 mm) from Phenomenex (CA, USA) and the mobile phase was composed of 0.1% formic acid in ultrapure water and a mixture of methanol and acetonitrile (7% v/v) with gradient program. The flow rate was set at 1.0 ml/min and the run time for each sample was 6 min.

### Immunophenotyping

Immunophenotyping was performed on cryopreserved PBMCs as previously described^53^. Briefly, 1 × 10^6^ cells were stained with 2 panels of antibodies (all from BD Pharmigen, San Jose, California, USA): (1) CD3-PerCP-Cy5.5, CD4-PE-Cy7, CD8-APC-H7, CD28-APC, CD57-FITC, CD45RA-PE, CCR7-BV421 and Fixable viability stain 510 (FVS510); and (2) CD3, CD4, CD8, CD38-PE, HLA-DR-BV421, CD25-BB515 and viability stain. Cells in the second panel were further fixed, permeabilised with the Foxp3 staining buffer kit (BD Pharmigen, CA, USA) and stained for anti-Foxp3-AF 647(clone 259D/C7) or IgG1 isotype control (Alexa Fluor 647; clone MOP-21). All cells were acquired on a BD FACS Canto II (BD Biosciences, CA, USA) and analysed using FACS Diva V6 (BD Biosciences, CA, USA). Following exclusion of doublets and dead cells, CD3+ cells were sequentially gated for CD4+ and CD8+ cells, followed by markers to identify maturational subsets (naïve: CCR7+CD45RA+; central memory, CM: CCR7+CD45RA−; effector memory, EM: CCR7-CD45RA-, terminally differentiated effector memory, TDEM: CCR7-CD45RA+); activation (CD38+HLA-DR+), senescence (CD28-CD57+) and T-regulatory cells (CD25+FOXP3+). See Supplementary Fig. S1 for detailed gating strategy.

### Statistical Analyses

The chi-squared test was used to test associations between categorical variables (i.e. between smoking status and control/HIV infected group). Where the result of chi-squared test was uncertain (expected cell count below 5), we used Fisher’s exact test. The non-parametric Mann-Whitney test was used to test whether the median of the continuous predictor variables differed significantly between control/HIV subgroups. Correlation between the bacterial taxa of interest and markers of immune activation and CD4 T-cell maturational subsets in HIV-infected participants was evaluated using Spearman’s rank correlation. Statistical significance was declared when p-value < 0.05. Canonical correlation analysis (CCA) was used to discover associations between two sets of variables, one consisting of CD4 T-cell count and CD4 T-cell subsets/markers of activation, and another consisting of selected bacterial taxa that differ significantly in relative abundance between the optimal and the suboptimal responders as obtained from LEfSe analysis. After identifying the correlated variables from CCA, multiple linear regression was used to retest the association between the relative abundance of the specific bacterial taxa and CD4 T-cell counts controlling for the effect of age, baseline CD4 T-cell counts, MSM status and antibiotic use. All statistical analyses were performed using R (Version 3.3.3)^62^.

## Electronic supplementary material

Supplemental Methods, Figures and Tables

## Data Availability

The datasets generated during and/or analysed during the current study are available in NCBI BioProject Repository (Accession Number: PRJNA489590).

## References

[1] Guadalupe M (2003). Severe CD4+ T-cell depletion in gut lymphoid tissue during primary human immunodeficiency virus type 1 infection and substantial delay in restoration following highly active antiretroviral therapy. J. Virol..

[2] Brenchley JM (2004). CD4+ T cell depletion during all stages of HIV disease occurs predominantly in the gastrointestinal tract. J. Exp. Med..

[3] Mehandru S (2004). Primary HIV-1 infection is associated with preferential depletion of CD4+ T lymphocytes from effector sites in the gastrointestinal tract. J. Exp. Med.

[4] Mehandru S (2007). Mechanisms of gastrointestinal CD4+ T-cell depletion during acute and early human immunodeficiency virus type 1 infection. J. Virol..

[5] Mehandru S, Tenner-Racz K, Racz P, Markowitz M (2005). The gastrointestinal tract is critical to the pathogenesis of acute HIV-1 infection. J. Allergy Clin. Immunol..

[6] Gordon SN (2010). Disruption of intestinal CD4+ T cell homeostasis is a key marker of systemic CD4+ T cell activation in HIV-infected individuals. J. Immunol..

[7] Chun TW (2008). Persistence of HIV in gut-associated lymphoid tissue despite long-term antiretroviral therapy. J. Infect. Dis..

[8] Yukl SA (2010). Differences in HIV burden and immune activation within the gut of HIV-positive patients receiving suppressive antiretroviral therapy. J. Infect. Dis..

[9] Yukl SA (2010). Effect of raltegravir-containing intensification on HIV burden and T-cell activation in multiple gut sites of HIV-positive adults on suppressive antiretroviral therapy. AIDS.

[10] Maidji E, Somsouk M, Rivera JM, Hunt PW, Stoddart CA (2017). Replication of CMV in the gut of HIV-infected individuals and epithelial barrier dysfunction. PLoS Pathog..

[11] Brenchley JM (2006). Microbial translocation is a cause of systemic immune activation in chronic HIVinfection. Nat. Med..

[12] Sandler NG (2011). Plasma levels of soluble CD14 independently predict mortality in HIV infection. J. Infect. Dis..

[13] Serrano-Villar S, Ferrer M, Gosalbes MJ, Moreno S (2017). How can the gut microbiota affect immune recovery in HIV-infected individuals?. Future Microbiol..

[14] Serrano-Villar S (2016). Gut Bacteria Metabolism Impacts Immune Recovery in HIV-infected Individuals. EBioMedicine.

[15] Vazquez-Castellanos JF (2015). Altered metabolism of gut microbiota contributes to chronic immune activation in HIV-infected individuals. Mucosal Immunol..

[16] Noguera-Julian M (2016). Gut Microbiota Linked to Sexual Preference and HIV Infection. EBioMedicine.

[17] Nowak P (2015). Gut microbiota diversity predicts immune status in HIV-1 infection. AIDS.

[18] Perez-Santiago J (2013). Gut Lactobacillales are associated with higher CD4 and less microbial translocation during HIV infection. AIDS.

[19] Dillon SM (2014). An altered intestinal mucosal microbiome in HIV-1 infection is associated with mucosal and systemic immune activation and endotoxemia. Mucosal Immunol..

[20] Vujkovic-Cvijin I (2013). Dysbiosis of the gut microbiota is associated with HIV disease progression and tryptophan catabolism. Sci. Transl. Med..

[21] Mutlu EA (2014). A compositional look at the human gastrointestinal microbiome and immune activation parameters in HIV infected subjects. PLoS Pathog..

[22] Dinh DM (2015). Intestinal microbiota, microbial translocation, and systemic inflammation in chronic HIV infection. J. Infect. Dis..

[23] Gori A (2011). Specific prebiotics modulate gut microbiota and immune activation in HAART-naive HIV-infected adults: results of the “COPA” pilot randomized trial. Mucosal Immunol..

[24] Klatt NR (2013). Probiotic/prebiotic supplementation of antiretrovirals improves gastrointestinal immunity in SIV-infected macaques. J. Clin. Invest..

[25] Stiksrud B (2015). Reduced Levels of D-dimer and Changes in Gut Microbiota Composition After Probiotic Intervention in HIV-Infected Individuals on Stable ART. J. Acquir. Immune Defic. Syndr..

[26] Miller H, Ferris R, Phelps BR (2016). The effect of probiotics on CD4 counts among people living with HIV: a systematic review. Beneficial microbes.

[27] Vujkovic-Cvijin Ivan, Rutishauser Rachel L., Pao Montha, Hunt Peter W., Lynch Susan V., McCune Joseph M., Somsouk Ma (2017). Limited engraftment of donor microbiome via one-time fecal microbial transplantation in treated HIV-infected individuals. Gut Microbes.

[28] Hensley-McBain T (2016). Effects of Fecal Microbial Transplantation on Microbiome and Immunity in Simian Immunodeficiency Virus-Infected Macaques. J. Virol..

[29] Miller GE (2016). Lower Neighborhood Socioeconomic Status Associated with Reduced Diversity of the Colonic Microbiota in Healthy Adults. PLoS One.

[30] Yatsunenko T (2012). Human gut microbiome viewed across age and geography. Nature.

[31] Monaco CL (2016). Altered Virome and Bacterial Microbiome in Human Immunodeficiency Virus-Associated Acquired Immunodeficiency Syndrome. Cell Host Microbe.

[32] Sun Y (2016). Fecal bacterial microbiome diversity in chronic HIV-infected patients in China. Emerg Microbes Infect.

[33] Nowak RG (2017). Rectal microbiota among HIV-uninfected, untreated HIV, and treated HIV-infected in Nigeria. AIDS.

[34] Lozupone CA (2013). Alterations in the gut microbiota associated with HIV-1 infection. Cell Host Microbe.

[35] McHardy IH (2013). HIV Infection is associated with compositional and functional shifts in the rectal mucosal microbiota. Microbiome.

[36] Wu JR (2013). Mycoplasmas infection in male HIV/AIDS patients in Jiangsu, China. Microb. Pathog..

[37] Yu G, Fadrosh D, Ma B, Ravel J, Goedert JJ (2014). Anal microbiota profiles in HIV-positive and HIV-negative MSM. AIDS.

[38] Piconi S (2010). Immune activation, apoptosis, and Treg activity are associated with persistently reduced CD4+ T-cell counts during antiretroviral therapy. AIDS.

[39] Scully C (1999). Periodontopathic bacteria in English HIV-seropositive persons. AIDS Patient Care STDS.

[40] Gonzalez OA, Li M, Ebersole JL, Huang CB (2010). HIV-1 reactivation induced by the periodontal pathogens *Fusobacterium* nucleatum and Porphyromonas gingivalis involves Toll-like receptor 2 [corrected] and 9 activation in monocytes/macrophages. Clin. Vaccine Immunol..

[41] Tahara T (2015). *Fusobacterium* detected in colonic biopsy and clinicopathological features of ulcerative colitis in Japan. Dig. Dis. Sci..

[42] Lee Y, Eun CS, Lee AR, Park CH, Han DS (2016). *Fusobacterium* Isolates Recovered From Colonic Biopsies of Inflammatory Bowel Disease Patients in Korea. Ann. Lab. Med..

[43] Tahara, T. *et al*. Potential link between *Fusobacterium* enrichment and DNA methylation accumulation in the inflammatory colonic mucosa in ulcerative colitis. *Oncotarget*, 10.18632/oncotarget.18716 (2017). 10.18632/oncotarget.18716 PMC5617474 28977914

[44] Swidsinski A (2011). Acute appendicitis is characterised by local invasion with *Fusobacterium* nucleatum/necrophorum. Gut.

[45] Rubinstein MR (2013). *Fusobacterium* nucleatum promotes colorectal carcinogenesis by modulating E-cadherin/beta-catenin signaling via its FadA adhesin. Cell Host Microbe.

[46] Kostic AD (2013). *Fusobacterium* nucleatum potentiates intestinal tumorigenesis and modulates the tumor-immune microenvironment. Cell Host Microbe.

[47] Imai K, Yamada K, Tamura M, Ochiai K, Okamoto T (2012). Reactivation of latent HIV-1 by a wide variety of butyric acid-producing bacteria. Cell. Mol. Life Sci..

[48] Jewett A (2000). Induction of apoptotic cell death in peripheral blood mononuclear and polymorphonuclear cells by an oral bacterium, *Fusobacterium* nucleatum. Infect. Immun..

[49] Shaw JM (2011). Increased frequency of regulatory T cells accompanies increased immune activation in rectal mucosae of HIV-positive noncontrollers. J. Virol..

[50] Mendez-Lagares G (2012). Severe immune dysregulation affects CD4(+)CD25(hi)FoxP3(+) regulatory T cells in HIV-infected patients with low-level CD4 T-cell repopulation despite suppressive highly active antiretroviral therapy. J. Infect. Dis..

[51] Favre D (2010). Tryptophan catabolism by indoleamine 2,3-dioxygenase 1 alters the balance of TH17 to regulatory T cells in HIV disease. Sci. Transl. Med..

[52] Yap SH (2017). HIV/Human herpesvirus co-infections: Impact on tryptophan-kynurenine pathway and immune reconstitution. PLoS One.

[53] Leng CY (2017). Human papillomavirus 16 (HPV16) and HPV52 E6-specific immunity in HIV-infected adults on combination antiretroviral therapy. HIV Med..

[54] Caporaso JG (2011). Global patterns of 16S rRNA diversity at a depth of millions of sequences per sample. Proc. Natl. Acad. Sci. USA.

[55] Chua LL (2017). Reduced microbial diversity in adult survivors of childhood acute lymphoblastic leukemia and microbial associations with increased immune activation. Microbiome.

[56] Caporaso JG (2010). QIIME allows analysis of high-throughput community sequencing data. Nat Methods.

[57] Chen Li, Reeve James, Zhang Lujun, Huang Shengbing, Wang Xuefeng, Chen Jun (2018). GMPR: A robust normalization method for zero-inflated count data with application to microbiome sequencing data. PeerJ.

[58] Segata N (2011). Metagenomic biomarker discovery and explanation. Genome Biol..

[59] Benjamini Y, Hochberg Y (1995). Controlling the False Discovery Rate - a Practical and Powerful Approach to Multiple Testing. J Roy Stat Soc B Met.

[60] Rajasuriar R (2015). The CD14 C-260T single nucleotide polymorphism (SNP) modulates monocyte/macrophage activation in treated HIV-infected individuals. J. Transl. Med..

[61] Huang Y (2013). A simple LC-MS/MS method for determination of kynurenine and tryptophan concentrations in human plasma from HIV-infected patients. Bioanalysis.

[62] Team RC. R: A language and environment for statistical computing. R Foundation for Statistical Computing, Vienna, Austria (2017).

