# Supplementary material for: Enrichment of gut-derived Fusobacterium is associated with suboptimal immune recovery in HIV-infected individuals
Source: Sci Rep. 2018 Sep 24;8:14277. doi: 10.1038/s41598-018-32585-x (PMC6155144; doi:10.1038/s41598-018-32585-x)
Supplement: Supplementary file 1 — Supplemental Methods, Figures and Tables [file 41598_2018_32585_MOESM1_ESM.pdf]

Enrichment of gut-derived *Fusobacterium* is associated with suboptimal immune recovery in HIV-infected individuals

Soo Ching Lee[1,2], Ling Ling Chua[3], Siew Hwei Yap[1], Tsung Fei Khang[4,5], Chan Yoon Leng[1], Iskandar Azwa[1,6], Sharon R Lewin[7,8], Adeeba Kamarulzaman[1,6], Yin Ling Woo[1,3,9], Yvonne Ai Lian Lim[1,2], P'ng Loke[10,*], Reena Rajasuriar[1,7,11,*]

* Combined last/corresponding authors

[1]Centre of Excellence for Research in AIDS (CERiA), University of Malaya, 50603 Kuala Lumpur, Malaysia,

[2]Department of Parasitology, Faculty of Medicine, University of Malaya, 50603 Kuala Lumpur, Malaysia,

[3]University Malaya Cancer Research Institute, University of Malaya, 50603 Kuala Lumpur, Malaysia,

[4]Institute of Mathematical Sciences, Faculty of Science, University of Malaya, 50603 Kuala Lumpur, Malaysia,

[5]University of Malaya Centre for Data Analytics, University of Malaya, 50603 Kuala Lumpur, Malaysia

[6]Department of Medicine, Faculty of Medicine, University of Malaya, 50603 Kuala Lumpur, Malaysia,

[7]Peter Doherty Institute for Infection and Immunity, University of Melbourne, Melbourne, Victoria, Australia,

[8]Department of Infectious Diseases, Monash University and Alfred Hospital; Royal Melbourne Hospital, Melbourne Australia

[9]Department of Obstetrics and Gynecology, Faculty of Medicine, University of Malaya, Kuala Lumpur, Malaysia.

[10]Department of Microbiology and Medicine, New York University School of Medicine, New York, NY 10016, USA,

[11]Department of Pharmacy, Faculty of Medicine, University of Malaya, 50603 Kuala Lumpur, Malaysia.

Correspondence to:

1) Reena Rajasuriar, Department of Pharmacy, Faculty of Medicine, 50603 Kuala Lumpur Malaysia; Tel: 603-79676617; email: reena@um.edu.my;

2) P'ng Loke, Department of Microbiology and Medicine, New York University School of Medicine, New York, NY 10016, USA; Tel: 1(646)501-4649; email: Png.Loke@nyumc.org

Supplemental Methods

Gut microbiota bioinformatics analysis. The paired end raw sequencing reads were joined using fastq-join function from EA-utils[1] under the default parameters of QIIME. Then, the sequences were demultiplexed and quality filtered using the default values of the split_libraries_fastq.py script. Subsequently, demultiplexed reads were clustered in 97% using the default UCLUST algorithm[2] open-reference OTU picking (pick_open_reference_otus.py) workflow based on Greengenes 13_8 database and PyNAST[3] for alignment. Taxonomy was assigned to sequences with the UCLUST consensus taxonomy assigner. In addition, chimera sequences were identified and removed using ChimeraSlayer algorithm within QIIME[4]. Then, phylogenetic tree was constructed using FastTree[5] alignment algorithm for downstream analysis. In this study, reads were successfully clustered into 14,362 operational taxonomic units (OTUs) with 97% identity.

Downstream analysis including taxa summary plot, alpha diversity and beta diversity were performed using QIIME. The relative abundance of all OTUs in the samples were averaged across the HIV-infected (i.e. HIV optimal and HIV suboptimal) and uninfected samples. Alpha rarefaction curves were computed by calculating the metrics observed OTUs, Phylogenetic distance[6] and Shannon index[7] using alpha_rarefration.py. The nonparametric t-test was done using compare_alpha_diversity.py script and the results were corrected by bonferroni test. Beta diversity was calculated using beta_diversity_through_plots.py. UniFrac[8] weighted and unweighted were then performed to determine the similarity among the microbial communities and visualized using principal coordinate analysis (PCoA) plots. Subsequently, the non-parametric multivariate analysis of variance with permutation (PERMANOVA), which uses the

unweighted and weighted UniFrac metric to measure phylogenetic distance, was used to test whether between group gut microbiota composition (HIV-infected vs. non-infected & oIR vs. sIR) differred significantly from within group composition.

References

1 Aronesty, E. *ea-utils*. " Commnad-line tools for processing biological sequencing data". (2011).

2 Edgar, R. C. Search and clustering orders of magnitude faster than BLAST. *Bioinformatics* **26**, 2460-2461, doi:10.1093/bioinformatics/btq461 (2010).

3 Caporaso, J. G. *et al.* PyNAST: a flexible tool for aligning sequences to a template alignment. *Bioinformatics* **26**, 266-267, doi:10.1093/bioinformatics/btp636 (2010).

4 Haas, B. J. *et al.* Chimeric 16S rRNA sequence formation and detection in Sanger and 454-pyrosequenced PCR amplicons. *Genome Res.* **21**, 494-504, doi:10.1101/gr.112730.110 (2011).

5 Price, M. N., Dehal, P. S. & Arkin, A. P. FastTree: computing large minimum evolution trees with profiles instead of a distance matrix. *Mol. Biol. Evol.* **26**, 1641-1650, doi:10.1093/molbev/msp077 (2009).

6 Faith, D. P. & Baker, A. M. Phylogenetic diversity (PD) and biodiversity conservation: some bioinformatics challenges. *Evol Bioinform Online* **2**, 121-128 (2007).

7 Shannon, C. E. The mathematical theory of communication. 1963. *MD Comput.* **14**, 306-317 (1997).

8 Lozupone, C. & Knight, R. UniFrac: a new phylogenetic method for comparing microbial communities. *Appl. Environ. Microbiol.* **71**, 8228-8235, doi:10.1128/AEM.71.12.8228-8235.2005 (2005).

Figure Legends

Supplementary Fig. S1. Flow cytometry gating strategy. Panel A: Following gating to exclude doublets, CD3+ T-cells were sequentially gated for CD4+ and CD8+ cells. In each of these subsets, the proportion of maturational subsets were identified as follows; naïve (CD45RA+CCR7+), central memory, CM (CD45RA-CCR7+), effector memory, EM (CD45RA-CCR7-) and terminally-differentiated effector memory, TdEM (CD45RA+CCR7-). Panel B: CD3+ cells were sequentially gated for CD4+ and CD8+ T-cells and subsequently markers of activation (CD38+HLA-DR+) and senescence (CD57+CD28-). T-regulatory cells were reported as the proportion of CD3+CD4+ T-cells expressing CD25+Foxp3+.

Supplementary Fig. S2. The top relative abundance of **a)** phyla and **b)** genus across the 46 subjects from the uninfected, HIV optimal and HIV suboptimal groups. The HIV positive samples were arranged according to increasing of CD4 T-cell counts. Histograms are based on the proportion of OTUs per subject.

Supplementary Fig. S3. PCoA plots assessing the beta diversity of microbial communities in i) HIV-infected and uninfected using **a)** unweighted and **b)** weighted UniFrac distance and ii) HIV suboptimal and HIV optimal using **c)** unweighted and **d)** weighted UniFrac distance. PERMANOVA was performed to determine the statistical significance. $p<0.05$ is considered significant.

Supplementary Fig. S4. Rarefraction curves assessing alpha diversity was plotted for **a)** Observed OTUs, **b)** Phylogenetic distance and **c)** Shannon index. There was no significant difference between the groups tested when using T test, p>0.05 for all comparisons.

Supplementary Fig. S5. Differences in the abundance of bacterial communities between the HIV-infected and uninfected groups. **a)** The uninfected group-enriched taxa are displayed with a positive LDA score (green) while the HIV-infected-enriched taxa are shown with a negative LDA score (red). Only taxa meeting an LDA score threshold >3 are listed**. b)** Bacterial taxa that were differentially abundant in the gut microbiota profiles between the HIV-infected and uninfected subjects visualized using a cladogram generated from LEfSe analysis. **c)** Scatter plot illustrating the association between the relative abundance of *Fusobacterium* and CD4 T-cell counts among the HIV-infected subjects.

Supplementary Table S1. Bacteria taxa which were different in abundance between HIV-infected and uninfected, after Benjamini-Hochberg correction.

**Taxonomic Hierarchy**	**Enriched in Group**	**Log LDA score**	**p-value**	**q-value**
Bacteria|p__Fusobacteria	HIV positive	4.505	0.0022	0.0154
Bacteria|p__Fusobacteria|c__Fusobacteriia	HIV positive	4.505	0.0022	0.0154
Bacteria|p__Fusobacteria|c__Fusobacteriia|o__Fusobacteriales	HIV positive	4.505	0.0022	0.0154
Bacteria|p__Fusobacteria|c__Fusobacteriia|o__Fusobacteriales|f__ Fusobacteriaceae	HIV positive	4.501	0.0032	0.0154
Bacteria|p__Fusobacteria|c__Fusobacteriia|o__Fusobacteriales|f__ Fusobacteriaceae|g__*Fusobacterium*	HIV positive	4.500	0.0032	0.0154
Bacteria|p__Firmicutes|c__Clostridia|o__Clostridiales|f__Veillone llaceae	HIV positive	4.211	0.0157	0.0326
Bacteria|p__Firmicutes|c__Clostridia|o__Clostridiales|f__Veillone llaceae|g__Megamonas	HIV positive	4.063	0.0024	0.0154
Bacteria|p__Proteobacteria|c__Gammaproteobacteria|o__Aeromo nadales|f__Succinivibrionaceae	HIV positive	3.952	0.0248	0.0421
Bacteria|p__Proteobacteria|c__Gammaproteobacteria|o__Aeromo nadales|f__Succinivibrionaceae|g__*Succinivibrio*	HIV positive	3.941	0.0355	0.0450
Bacteria|p__Proteobacteria|c__Gammaproteobacteria|o__Aeromo nadales	HIV positive	3.940	0.0144	0.0326
Bacteria|p__Tenericutes|c__Mollicutes|o__Mycoplasmatales	HIV positive	3.300	0.0403	0.0450
Bacteria|p__Tenericutes|c__Mollicutes|o__Mycoplasmatales|f__ Mycoplasmataceae	HIV positive	3.275	0.0403	0.0450
Bacteria|p__Actinobacteria|c__Actinobacteria|o__Actinomycetale s|f__Actinomycetaceae|g__*Mobiluncus*	HIV positive	3.037	0.0253	0.0421

Bacteria|p__Proteobacteria|c__Betaproteobacteria|o__Neisseriales |f__Neisseriaceae	Uninfected	3.688	0.0324	0.0448
Bacteria|p__Proteobacteria|c__Betaproteobacteria|o__Neisseriales	Uninfected	3.660	0.0324	0.0448
Bacteria|p__Firmicutes|c__Erysipelotrichi|o__Erysipelotrichales|f __Erysipelotrichaceae|g___*Eubacterium*_	Uninfected	3.283	0.0376	0.0450
Bacteria|p__Bacteroidetes|c__Bacteroidia|o__Bacteroidales|f___B arnesiellaceae_	Uninfected	3.238	0.0395	0.0450
Bacteria|p__Firmicutes|c__Clostridia|o__Clostridiales|f__Rumino coccaceae|g__*Oscillospira*	Uninfected	3.180	0.0261	0.0421
Bacteria|p__Synergistetes|c__Synergistia|o__Synergistales|f__Det hiosulfovibrionaceae|g__*Jonquetella*	Uninfected	3.145	0.0435	0.0451
Bacteria|p__Firmicutes|c__Clostridia|o__Clostridiales|f__Lachnos piraceae|g__*Moryella*	Uninfected	3.082	0.0435	0.0451

Supplementary Table S2. Bacteria taxa which were different in abundance between HIV optimal (oIR) and HIV Suboptimal (sIR) arms, after Benjamini-Hochberg correction.

**Taxonomic Hierarchy**	**Enriched in Group**	**Log LDA score**	**p-value**	**q-value**
Bacteria|p__Firmicutes|c__Bacilli|o__Lactobacillales	HIV optimal	4.432	0.0307	0.0208
Bacteria|p__Actinobacteria|c__Actinobacteria|o__Actinomycetales|f__Corynebacteriaceae|g__*Corynebacterium*	HIV optimal	4.340	0.0452	0.0292
Bacteria|p__Actinobacteria|c__Actinobacteria|o__Actinomycetales|f__Corynebacteriaceae	HIV optimal	4.340	0.0452	0.0313
Bacteria|p__Fusobacteria|c__Fusobacteriia|o__Fusobacteriales|f__Fusobacteriaceae	HIV suboptimal	4.695	0.0424	0.0229
Bacteria|p__Fusobacteria|c__Fusobacteriia|o__Fusobacteriales|f__Fusobacteriaceae|g__*Fusobacterium*	HIV suboptimal	4.695	0.0424	0.0250
Bacteria|p__Fusobacteria	HIV suboptimal	4.688	0.0481	0.0458
Bacteria|p__Fusobacteria|c__Fusobacteriia	HIV suboptimal	4.688	0.0481	0.0479
Bacteria|p__Firmicutes|c__Clostridia|o__Clostridiales|f___Tissierellaceae_|g__*Gallicola*	HIV suboptimal	3.158	0.0073	0.0042
Bacteria|p__Proteobacteria|c__Deltaproteobacteria|o__Desulfovibrionales	HIV suboptimal	3.093	0.0479	0.0375
Bacteria|p__Proteobacteria|c__Deltaproteobacteria	HIV suboptimal	3.093	0.0479	0.0396
Bacteria|p__Proteobacteria|c__Deltaproteobacteria|o__Desulfovibrionales|f__Desulfovibrionaceae	HIV suboptimal	3.093	0.0479	0.0417
Bacteria|p__Proteobacteria|c__Deltaproteobacteria|o__Desulfovibrionales|f__Desulfovibrionaceae|g__*Bilophila*	HIV suboptimal	3.075	0.0479	0.0438

Supplementary Table S3. Results of CCA showing the associations between CD4 and CD8 T-cell counts and selected bacterial taxa found most abundantly different in LEfSe analysis in optimal and suboptimal immune responders.

	**Total Samples (N=26)**
	**Canonical Variate 1**
**Variables**	**Canonical Loadings**
**Immune Cells**	
CD4 count	0.846
CD8 count	-0.051
**Gut microbiota**	
g_*Bilophila*	-0.113
g_*Gallicola*	-0.103
g_*Corynebacterium*	0.437
g_*Fusobacterium*	-0.691
o_Lactobacillales	0.582
Aggregate Redundancy Coefficients	
Immune cells | Gut microbiota	0.300
Gut microbiota | Immune cells	0.162

Supplementary Table S4. Results of CCA showing the associations between CD4 T-cell maturational subsets and selected bacterial taxa found significantly associated with CD4 T-cell counts in HIV-infected participants.

	**Total Samples (N=26)**
	**Canonical Variate 1**
**Variables**	**Canonical Loadings**
**Immune Cells**	
Naïve CD4	0.509
CD4 Activation	-0.923
CD4 Tregs	-0.639
**Gut microbiota**	
g_*Corynebacterium*	-0.199
g_*Fusobacterium*	-0.951
o_Lactobacillales	0.361
Aggregate Redundancy Coefficients	
Immune cells | Gut microbiota	0.096
Gut microbiota | Immune cells	0.092
